# Gene polymorphisms associated with immunosuppressant adverse effects in systemic lupus erythematosus: a narrative review

**DOI:** 10.3389/fgene.2025.1594648

**Published:** 2025-06-24

**Authors:** Siva Hamdani, Laniyati Hamijoyo, Riezki Amalia, Melisa I. Barliana

**Affiliations:** ^1^ Department of Biological Pharmacy, Faculty of Pharmacy, Universitas Padjadjaran, Sumedang, Indonesia; ^2^ Unit of Pharmacology, Clinical Pharmacy and Community, Faculty of Science, Universitas Garut, Garut, Indonesia; ^3^ Division of Rheumatology, Department of Internal Medicine, Faculty of Medicine, Universitas Padjadjaran, Hasan Sadikin Hospital, Bandung, Indonesia; ^4^ Department of Pharmacology and Clinical Pharmacy, Faculty of Pharmacy, Universitas Padjadjaran, Bandung, Indonesia; ^5^ Center of Excellence for Pharmaceutical Care Innovation, Universitas Padjadjaran, Bandung, Indonesia

**Keywords:** single nucleotide polymorphism, adverse effect, methotrexate, azathioprine, cyclophosphamide, mycophenolate mofetil

## Abstract

Systemic Lupus Erythematosus (SLE) is an autoimmune disease that often requires treatment with immunosuppressant drugs to manage symptoms and prevent organ damage. However, the use of immunosuppressant can be associated with various adverse effects. The spectrum of immunosuppressant toxicity is influenced by various factors such as organ function and medication interval, but genetic variations—particularly single nucleotide polymorphisms—have emerged as critical determinants due to their direct impact on the drug’s pharmacokinetics and pharmacodynamics alteration, also on patient susceptibility to adverse reactions. This review summarizes the current knowledge on gene polymorphisms associated with immunosuppressant adverse effects in SLE patients, focusing on commonly used drugs such as Methotrexate (MTX), Azathioprine (AZA), Cyclophosphamide (CYC), and Mycophenolate Mofetil (MMF). A total of 23 relevant studies published in the last decade were identified through a comprehensive literature search, specifically investigating the relationship between gene polymorphisms and adverse drug reactions in SLE patients. The findings reveal that gene polymorphisms are frequently associated with adverse effects for each immunosuppressant, including MTX (*MTHFR* and *ATIC*), AZA (*TPMT, NUDT15, ITPA, ABCC4*), CYC (*CYP2C19, GSTM1, GSTT1, GSTP1, ALDH*), and MMF (*SLCO1B1, IMPDH1, UGT2B7*). Understanding the functional implications of these gene polymorphisms contributes to the application of precision medicine, as they can serve as potential markers for drug selection and dosage adjustment during initiation treatment of immunosuppressant to enhance treatment efficacy, minimize toxicity, and improve outcomes for SLE patients.

## 1 Introduction

Systemic Lupus Erythematosus (SLE) is a chronic autoimmune inflammatory disease featuring complex pathogenesis that affects various organ systems, leading to significant mortality and morbidity (Basta et al., 2020; Cattaneo et al., 2008). The incidence and prevalence of SLE vary widely across different ethnicities and regions, including the United States, Europe, the Middle East, and Asia (Ju et al., 2016). Treatment management strategies depend on disease type and severity, with mild to moderate cases typically treated using nonsteroidal anti-inflammatory drugs (NSAIDs), antimalarial agents (e.g., hydroxychloroquine), and corticosteroids. As the disease severity increases, high-dose corticosteroids and immunosuppressive agents, including Methotrexate (MTX), azathioprine (AZA), cyclophosphamide (CYC), and mycophenolate mofetil (MMF), are often used to control symptoms. The selection of immunosuppressant in SLE patients depends on disease manifestations, organ involvement, patient age, childbearing potential, safety considerations, and cost. However, their clinical utility is frequently constrained by serious adverse effects due to their narrow therapeutic index. These adverse effects not only increase the risk of long-term organ damage and treatment failure but also contribute to higher mortality rates and reduced quality of life, even during remission phases (Basta et al., 2020).

Among the many factors influencing immunosuppressant-related serious adverse effects, genetic variability—particularly in the form of single nucleotide polymorphisms (SNPs)—has gained considerable attention. These genetic differences can affect drug metabolism, efficacy, and the likelihood of adverse effects. Pharmacogenomics, the study of the role of genetics in drug response, was introduced to optimize the treatment while minimizing drug-related toxicity using SNPs as molecular markers, forming the basis for precision medicine (Cattaneo et al., 2008). A growing number of pharmacogenomic studies in SLE have explored the role of SNPs in determining the safety and effectiveness of immunosuppressants (Petri et al., 2012). This narrative review aims to summarize and discuss the current knowledge on gene polymorphisms associated with adverse effects from the most commonly used immunosuppressants in SLE: MTX, AZA, CYC, and MMF. Although these agents are administered within relatively standardized therapeutic dose ranges, the incidence and severity of adverse effects vary significantly among individuals. This variability can be attributed, in part, to genetic polymorphisms that affect drug metabolism, transport, and cellular targets. Therefore, elucidating the role of genetic polymorphisms is essential for understanding the underlying mechanisms of immunosuppressant-induced adverse effects. By highlighting the associations between specific genetic variants and drug toxicities, this review provides a foundation for integrating genetic screening into clinical decision-making. Such an approach may enhance treatment efficacy and safety, ultimately leading to improved SLE patient outcomes. The subsequent sections of this review will explore each immunosuppressant in detail. For each drug, we will discuss its pharmacological mechanism, common adverse effects, and the genetic polymorphisms known to influence its toxicity. This structure is intended to offer a practical, drug-centered understanding of how pharmacogenetics can inform and refine SLE treatment.

## 2 Method

This manuscript is a narrative review article. The search strategy and inclusion criteria were conducted using Google Scholar and PubMed databases, which include the use of Boolean operators for keyword combinations. Specifically, these keywords were combined using Boolean operators (AND, OR) as follows: “genetic polymorphism” OR “SNPs” AND “immunosuppressant drugs” OR “azathioprine” OR “thiopurine” OR “methotrexate” OR “cyclophosphamide” OR “mycophenolate mofetil” OR “SLE therapy” AND “Adverse effects”. We included studies published in English, excluding narrative reviews, communication studies, and unpublished manuscripts. A total of 23 articles from the past 10 years were included in the review, categorized by drug: 7 on MTX, 8 on AZA, 6 on CYC, and 2 on MMF.

## 3 Gene polymorphisms and adverse effects of immunosuppressants in SLE

SLE is a highly diverse autoimmune condition, showing a wide array of symptoms and affecting various organs (Basta et al., 2020; Cattaneo et al., 2008; Ju et al., 2016; Petri et al., 2012; Hochberg, 1997). This disease arises when the immune system erroneously attacks healthy tissues, leading to inflammation and harm, such as visceral damage, flare-ups, neuropsychiatric lupus, and many more. The severity and specific expressions of SLE can differ significantly from one individual to another. Patients with mild SLE are primarily given a low dose of glucocorticoids (GCs) as therapy because of effectivity in controlling SLE activity rapidly and reducing exacerbation (Katarzyna et al., 2023). A higher dose of GCs is used during more severe SLE activity or in some life-threatening conditions, such as lupus nephritis (LN). However, the adverse effects of GCs are dose-dependent, suggesting that an increase in administered GCs dose leads to higher risk of adverse effects, such as infection, cardiovascular disease, cancer, osteoporosis, and many more (McKeon and Jiang, 2020). To mitigate these risks, combination therapy involving GCs and immunosuppressive agents is often employed. As reviewed in the literature, most patients receiving immunosuppressive treatment also remain on concurrent GC therapy to achieve synergistic therapeutic effects.

Immunosuppressant is used when the disease progresses from moderate to severe condition and the administration of GCs as SLE first-line treatment cannot sustain clinical remission (Gatto et al., 2019). In general, the immunosuppressant mechanism of action requires suppressing and decreasing the autoimmune responses, which can target various organs and systems in the body. This serves to minimize damage in various organs, thereby preventing life-threatening conditions. The use of the immunosuppressant is based on the organs engaged in SLE activity and the conditions of patients. Moderately active lupus and joints involved are treated with MTX, while LN and other severe cases are treated primarily with MMF and CYC (Mohamed et al., 2019; Fraenkel et al., 2021).

Immunosuppressant administration can decrease GCs exposure, stabilize SLE, and increase the probability of better survival than using GCs alone but toxicity incidence is high, ranging from 42.8% to 97.3% (Oglesby et al., 2013). Several immunosuppressive agents of the drugs can cause complications, such as liver dysfunction, bone marrow suppression, pulmonary toxicity, and many more. As the study of pharmacogenetics is advancing, variations in genomic diversity, including SNPs, are found to be a potential crucial factor affecting toxicity incidence due to the alteration of pharmacokinetics and pharmacodynamics of the administered drugs (Meng et al., 2018). The summary of all studies discussed, including the sample size, drug toxicity manifestation, and the most common gene polymorphisms associated with immunosuppressant adverse effects are shown in Table 1. Given the comparable spectrum of therapeutic doses and the potential for adverse reaction across diseases such as SLE, studies concerning genes implicated in the adverse effects of immunosuppressant drugs for treatment are also included.

**TABLE 1:** Summary of immunosuppressant-related adverse effects and gene polymorphisms.

No	Immunosuppre-ssants	Gene and mechanism type	Gene variant	Functional impact	Drug-related adverse effects and statistic value	Population and sample size	Reference
1	METHOTREXATE (MTX)	*MTHFR;* Pharmaco-dynamics	*MTHFR* c.667C>T (rs1801133)	Reduced MTHFR enzyme activity → disrupted folate metabolism and accumulation of homocysteine → exacerbate MTX’s antifolate effects, increasing the risk of adverse effects	TT genotype was significantly associated with higher MTX toxicity (OR = 1.615; 95% CI = 1.185–2.200) → GI reactions (Abdominal pain, diarrhea, N/V), Liver disease, Tiredness	Japan-China; 162 patients	Song et al. (2014)
2			*MTHFR* c.1298A>C (rs1801131)		CC genotype linked to highest MTX toxicity (83.3%; p = 0.003) → BMT, GI reactions (anorexia, nausea, vomiting, and diarrhea), mucocutaneous complaints (alopecia, rashes, and oral ulcers), CNS (insomnia, headache, and dizziness), hepatotoxicity	Egypt; 50 patients	Sharaki et al. (2018)
3			*MTHFR* c.667C>T (rs1801133) and c.1298A>C (rs1801131)		• No association was found between c.1298A>C polymorphism and MTX toxicity • T allele of the c.677C>T polymorphism was associated with the occurrence of MTX adverse effects (Nausea/Vomiting, Liver intolerance, Alopecia, Normocytic-normochromic anemia, Thrombopenia) (p = 0.019, OR: 3.63, 95% CI [1.12–12.80])	North India, 110 patients	Dwivedi et al. (2020)
4			*MTHFR* c.667C>T (rs1801133)		A higher risk of anemia (OR = 1.83), hepatotoxicity (OR = 1.98), neutropenia (OR = 2.2), and leukopenia (OR = 2.38)	China, 69 pediatric patients	Yang et al. (2023)
5		*ATIC;* Pharmaco-dynamics	*ATIC* c.347C>G (rs2372536) (dominant)	Reduced ATIC activity → Greater accumulation of AICAR → enhancing the anti-inflammatory action of MTX	Hepatotoxicity (p = 0.02; OR = 2.18)	Portugal; 44 patients	Martusevich et al. (2020)
6			*ATIC* c.347C>G (rs2372536)		GG + GC genotypes were associated with an increased risk of MTX toxicity (p = 0.032, Overall OR = 1.454, 95% CI [1.034–2.044]) → GI complaints, hepatotoxicity, BMT, dermatological complaints, lung toxicity, nervous system toxicity, kidney toxicity, infection and osteoporosis, fatigue, epistaxis	Caucasians, Asians; 6 patients	Lee and Bae. (2016)
7			*ATIC* c.347C>G (rs2372536)		GI complaints (N/V, diarrhea, *etc.*) OR = 4.46; 95% CI = 1.28–15.52; p = 0.02	South India; 319 patients	Muralidharan et al. (2016)
1	AZATHIOPRINE (AZA)	*TPMT;* Pharmaco-kinetics	TPMT*3C c.719A>G (rs1142345); TPMT*3B c.460G>A (rs1800460); TPMT*2 c.238G>C (rs1800462)	Reduced TPMT enzymatic activity → decreased inactivation of 6-MP → accumulation of active thioguanine nucleotides → myelosuppression	• TPMT*3C was associated with leukopenia grade III/IV (OR = 17.6; 95% CI = 5.8–53.6; p < 0.0001), and thrombocytopenia (OR = 13.4; 95% CI: 4.6–39.2; p < 0.0001) • TPMT*3B (rs1800460) and TPMT*2 were not associated with AZA’s adverse effects	Bangladesh; 250 patients	Rashid et al. (2020)
2			TPMT*3C c.719A>G (rs1142345)		Hepatotoxicity (HR = 3.85; 95% CI = 1.83–8.10; p = 0.0004)	Taiwan; 50 patients	Sheu et al. (2022)
3			TPMT*3 c.460G>A (rs1800460); TPMT*2 c.238G>C (rs1800462); TPMT*3 c.719A>G (rs1142345)		Anemia, leukopenia, and thrombocytopenia in • TPMT*3 c.460G>A (p = 0.173) • TPMT*2 c.238G>C (p = 0.032) • TPMT*3 c.719A>G (p = 0.153)	Egypt; 150 patients	Abuelsoud et al. (2021)
4		*NUDT15;* Pharmaco-kinetics	*NUDT15* c.415C>T (rs116855232) and TPMT*3C c.719A>G (rs1142345)	Reduced NUDT15 activity → accumulation of active thiopurine metabolites, decreased degradation of thioguanine nucleotides → increased DNA damage	• *NUDT15* c.415C>T → Leukopenia/neutropenia (OR = 1.08; 95% CI = 0.11–10.65; p = 0.95) • TPMT*3C c.719A>G → Leukopenia (OR = 7.59; 95% CI = 3.16–18.21; p < 0.0001)	China; 87 patients	Fei et al. (2018a)
5			NUDT15*3 c.415C>T (rs116855232)		Leukopenia (OR = 35.63; 95% CI = 22.47–56.51; p < 0.0001)	Korea; 978 patients	Yang et al. (2014)
			*NUDT15* c.415C>T (rs116855232); *NUDT15 55_56insGAGTCG* (rs746071566); TPMT*3C c.7196A>G (rs1142345)		Leukopenia • *NUDT15* c.415C>T (OR = 21.7; 95% CI = 12.1–38.8; p < 0.0001) • *NUDT15* 55_56insGAGTCG (OR = 7.1; 95% CI = 3.7–13.7; p < 0.0001) • TPMT*3C c.7196A>G (OR = 0.40; 95% CI = 0.0–6.8; p = 0.999)	China; 40 patients	Wang et al. (2022)
6		*ITPA;* Pharmacokinetics	TPMT*3A, TPMT*3B, TPMT*3C, TPMT*2*, ITPA* c.94C>A (rs1127354), *ITPA* c.124 + 21A>C (rs7270101)	Potentially reduced ITPA enzyme expression → accumulation of ITP/dITP in cells → **cellular damage**, particularly in hematopoietic tissues	• *TMPT* genes polymorphisms: Myelosuppression (p < 0.01) • No association between ITPA polymorphisms and AZA-related adverse effects	Lithuania; 551 patients	Steponaitiene et al. (2016)
7			*ITPA* c.94C>A (rs1127354); TPMT*3 (T>C), and *NUDT15* c.415C>T (rs116855232)	Reduced or absent ITPA activity → accumulation of ITP/dITP in cells → **cellular damage**, particularly in hematopoietic tissues	Myelosuppression • *ITPA* c.94C>A (OR = 4.945; 95% CI = 0.928–26.358; p = 0.061) • TPMT*3 (T>C) (OR = 0.00; 95% CI = −; p = 1.000) • *NUDT15* c.415C>T (OR = 51.818; 95% CI = 5.280–508.556; p = 0.001)	China; 1,419 patients	Chen et al. (2021)
8							
1	CYCLOPHOSPHAMIDE (CYC)	*GST;* Pharmacokinetics	*GSTM1* - (Null variant)*, GSTT1* - (Null variant), and *GSTP1 c.*313A>G (rs1695)	Reduced or complete absence enzyme activity → reduced the CYC metabolites detoxification efficiency → higher intercellular toxicity	• *GSTM1* - (Null variant): Nausea, rash, amenorrhea, diarrhea, neutropenia, infection (OR = 3.345; 95% CI = 1.064–10.577; p = 0.039) • *GSTP1* c.313A>G (rs1695): Independent factor of poor renal outcome (OR = 5.011; 95% CI = 1.025–24.510; p = 0.047) • *GSTT1* - (Null variant): No association	France; 70 patients	Audemard-Verger et al. (2016)
2		*CYP;* Pharmacokinetics	CYP2C19*2 c.681G>A (rs4244285)	Reduced enzyme activity → altered CYC metabolism → accumulation of toxic metabolites	Ovarian toxicity	India; 220 patients	Kumaraswami et al. (2017)
3			*CYP2B6* −750T>C (rs4802101); CYP2C19*2 c.681G>A (rs4244285); *GSTP1* c.313A>G (rs1695)		• *CYP2B6* -750T>C → GI toxicity (OR = 0.238; 95% CI = 0.107–0.523; p < 0.001); Leukocytopenia (OR = 0.347; 95% CI = 0.173–0.699; p < 0.001) • CYP2C19*2 c.681G>A → GI toxicity (OR = 0.157; 95% CI = 0.057–0.430; p < 0.001); Leukocytopenia (OR = 0.194; 95% CI = 0.092–0.409; p < 0.001); Infection (OR = 0.260; 95% CI = 0.119–0.568; p < 0.001) • *GSTP1* c.313A>G → OR = 1.791; 95% CI = 0.835–3.839; p > 0.05); Leukocytopenia (OR = 1.821; 95% CI = 0.953–3.482; p > 0.05)	China, 116 patients	Shu et al. (2016)
4		GSTP gene; Pharmacokinetics	*GSTP1* c.313A>G (rs1695)	lower catalytic efficiency → impact CYC detoxification capacity	Severe leukopenia (p < 0.05)	Indonesia; 91 patients	Hasni et al. (2016)
5		*ALDH;* Pharmacokinetics	*ALDH1A1* c.1234A>G (rs8187996)	reduced ALDH1A1 enzyme activity → affecting the detoxification process of cyclophosphamide metabolites	a reduced risk of severe toxicity (OR = 0.31; 95% CI = 0.09–0.78; p = 0.028) → contradictory findings	Michigan; 510 patients	Hwang et al. (2022)
6			ALDH3A1*1/*2 (rs2228100), ALDH3A1*2/*2 (rs2228100), ALDH1A1*2 (rs8187996)	*ALDH3A1* variants impairs aldehyde clearance → increasing the risk of hematologic and bladder toxicities *ALDH1A1* c.1234A>G (*rs8187996*) variant → reduced mRNA of *ALDH1A1* expression → reduced enzyme expression → higher risk of CYC toxicity	• ALDH3A1*2 (rs2228100) → Hemorrhagic cystitis (OR = 11.95; 95% CI = 1.18–120.56; p = 0.04); liver toxicity (OR = 5.13; 95% CI = 1.30–20.30; p = 0.02) • ALDH3A1*2/*2 (rs2228100) → Haemorrhagic cystitis (OR = 9.08; 95% CI = 1.02–80.58; p = 0.05) • ALDH1A1*2 (rs8187996) → Liver toxicity grade 3–4 (OR = 5.13; 95% CI = 1.30–20.30; p = 0.02)	Netherland; 113 patients	Ekhart et al. (2008)
1	MYCOPHENOLATE MOFETIL (MMF)	*IMPDH;* Pharmaco-dynamics	*IMPDH1* c.849T>G (rs2288553); c.662G>A (rs2288549); c.800G>A (rs2278293); −106G>A (rs2278294); and c.1575G>A (rs2228075)	SNPs of IMPDH1 are located in non-coding regions → alterations in gene expression, mRNA stability, or splicing efficiency → may alter the cellular sensitivity to MMF’s immunosuppressive action	GI Intolerance (p = 0.0005)	59 patients	Ohmann et al. (2010)
2		*SLCOB1;* Pharmacokinetics UGT2B7; Pharmacokinetics	*SLCO1B1* c.521T>C (rs4149056); *IMPDH1* −106G>A (rs2278294); *UGT2B7* c.802C>T (rs7439366)	*SLCO1B1* c.521T>C (rs4149056) → reduced transporter activity, leading to decreased hepatic uptake of MMF and consequently higher plasma concentrations; *UGT2B7* c.802C>T (rs7439366) → alter UGT2B7 enzyme activity which is involved in the glucuronidation and elimination of MMF	• *SLCO1B1* c.521T>C (rs4149056) → Anemia (p = 0.029) • *IMPDH1* −106G>A (rs2278294) → Infection (p = 0.006) • *UGT2B7* c.802C>T (rs7439366) → Pneumonia Infection (p = 0.036)	China; 120 patients	Shu et al. (2021)

### 3.1 Methotrexate

MTX is an antifolate antimetabolite commonly used in the treatment of RA, cancer, as well as SLE, and associated with a significant decrease in GCs dose used in adult patients. A systematic study showed that MTX appeared to offer significant benefits for individuals experiencing active arthritis or cutaneous symptoms in SLE (Sakthiswary and Suresh, 2014). The entry of MTX into the cell is facilitated by human reduced folate carriers (hFRC), a major importer of folates, known as SLC19A1. Following cellular uptake, MTX passes through polyglutamation by folylpolyglutamate synthase, leading to the retention in the cell. The mechanism of action includes folate metabolism, specifically inhibition of dihydrofolate reductase (DHFR), an enzyme essential in converting dihydrofolate to tetrahydrofolate (THF) active form. THF is indispensable for various cellular processes, including the synthesis of DNA and RNA nucleotides. MTX disrupts these processes by impeding DHFR, causing intracellular depletion of THF, particularly in rapidly dividing cells. Beyond DHFR inhibition, MTX and the polyglutamate forms impede *de novo* purine synthesis and thymidylate synthase, intensifying the cytotoxic effects that affect cell proliferation and growth (Mohamed et al., 2019).

**FIGURE 1:**
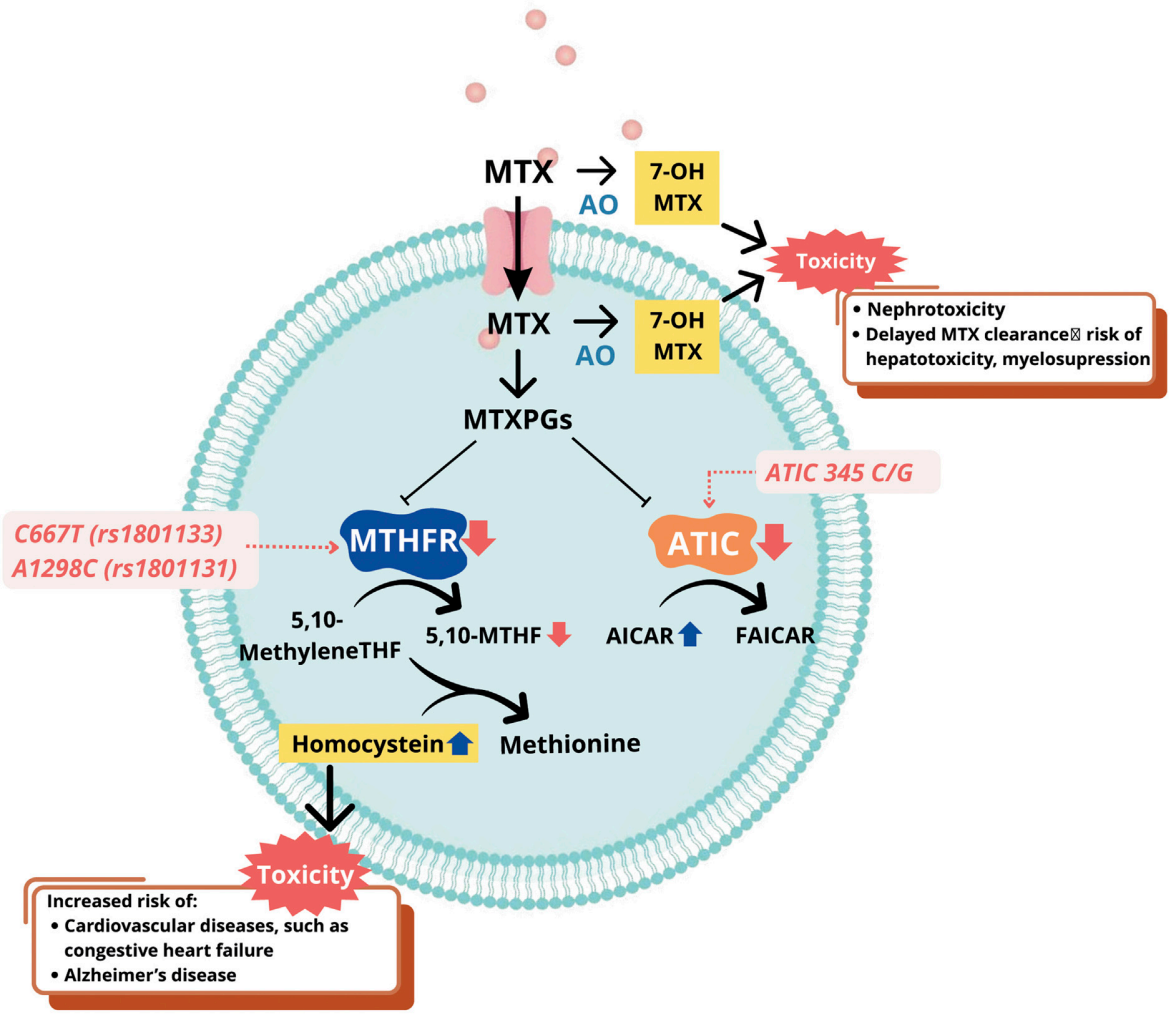
Mechanism of gene polymorphisms affecting MTX-related toxicity. Abbreviations: MTX, methotrexate; AO, aldehyde oxidase; 7-OH-MTX, 7-hydroxy methotrexate; ATIC, 5-aminoimidazole-4-carboxamide ribonucleotide formyltransferase/IMP cyclohydrolase; AICAR, aminoimidazole carboxamide ribonucleotide transformylase/IMP cyclohydrolase; MTHFR, methylenetetrahydrofolate reductase; 5,10-methylene THF, 5,10-methylenetetrahydrofolate; 5-THF, 5-methyltetrahydrofolate; MTXPGs, methotrexate polyglutamates; FAICAR, formyl-AICAR.

Despite the efficacy of MTX, related toxicity has been reported in several studies, including liver toxicity (increase of liver function, risk of liver failure), kidney toxicity (renal impairment, renal failure), hematological toxicity (pancytopenia, myelosuppression, leukopenia, neutropenia, megaloblastic anemia), pulmonary toxicity (wheezing, asthma), dermatological toxicity (skin lesion), and gastrointestinal (GI) effects (diarrhea, nausea, vomiting, *etc.*) (Hamed et al., 2022). The most common major toxicity of low-dose MTX was pancytopenia, followed by oral mucositis, hypoalbuminemia, acute renal failure, and pneumonitis, while minor toxicity included diarrhea, abdominal pain, and fever (Kivity et al., 2014). The duration of MTX use may affect the severity of the toxicity manifestation. Skin lesions and mucosal ulcers were reported in patients with less than 7 days of consumption, while more severe toxicity such as leukopenia, thrombocytopenia, and anemia were more common in those exposed to more than 7 days of consumption (Ahmadzadeh et al., 2019). Gene polymorphisms are suggested to affect the incidence of MTX-related toxicity, as several studies show that *MTHFR* c.667C>T, *MTHFR* c.1298A>C, and *ATIC* c.347C>G are the most common genes responsible for MTX toxicity, in both the treatment of SLE and other diseases including RA. Figure 1 presents effects of these gene polymorphisms on MTX-related toxicity.

#### 3.1.1 MTHFR polymorphisms

The *MTHFR* (Methylenetetrahydrofolate reductase) gene encodes a 77-kDa MTHFR enzyme which participates in folate metabolism, specifically in the conversion of 5,10-methylenetetrahydrofolate to 5-methyltetrahydrofolate (5-MTHF), and this process is crucial for the synthesis of nucleotides and DNA. Several studies, including meta-analyses, have reported an association between the two most common SNPs of the *MTHFR* gene, namely c.667C>T (rs1801133) and c.1298A>C (rs1801131), with MTX toxicity (Song et al., 2014; Dwivedi et al., 2020; Yang et al., 2023; Martusevich et al., 2020; Von Feldt et al., 2006; Juster-switlyk et al., 2017; Zhao et al., 2016; Campbell et al., 2016). In c.667C>T polymorphism, alanine is substituted with valine due to a change from C to T at nucleotide 677 (Rosenberg et al., 2002). Meanwhile, in c.1298A>C polymorphism, A is replaced with C at position 1,298, leading to the substitution of alanine to glutamine (Sharaki et al., 2018). Both SNPs lead to the lower enzymatic activity of MTHFR, affecting MTX pharmacodynamics (Van Der Put et al., 1998). Consequently, there is a decrease in the production of 5-MTHF, which serves as a methyl donor in the re-methylation of homocysteine to methionine (Figure 1). This leads to homocysteine accumulation in the blood, a condition known as hyperhomocysteinemia. Elevated homocysteine levels have been associated with increased toxicity of MTX and elevated cardiovascular risks, such as coronary artery calcification, high blood pressure, and many more (Von Feldt et al., 2006; Gande et al., 2023). The exact mechanism underlying MTX toxicity caused by lower MTHFR enzymatic activity remains unclear and requires further investigation.

#### 3.1.2 ATIC polymorphism

*ATIC* gene is located at chromosome 2q35 and encodes aminoimidazole carboxamide adenosine ribonucleotide transformylase (ATIC) that participates in the *de novo* purine synthesis and transforms aminoimidazole carboxamide adenosine ribonucleotide (AICAR) into formyl- AICAR. MTX mechanisms of action includes inhibiting ATIC after entering the cells, causing AICAR intracellular accumulation (Figure 1). This leads to the release of adenine into extracellular which inhibits the functions of several immune cells, such as monocytes, T-lymphocytes, and NK cells, initiating anti-inflammatory activities (Lee and Bae, 2016). The most commonly explored *ATIC* polymorphism is *ATIC* c.347C>G, with the C to G variation prompting the change of threonine to serine at position 116 of gene. Several studies showed that patients with the *ATIC* c.347G allele had a higher risk of MTX-related toxicity, specifically GI toxicity (Muralidharan et al., 2016; Grabar et al., 2010; Huang et al., 2020; Londono et al., 2020). Other studies on the same polymorphism did not report a relationship between gene and toxicity, but associated *ATIC* c.347C>G with the efficacy or non-responsiveness of MTX (Lee and Bae, 2016; Sha et al., 2022). A meta-analysis consisting of nine comparative studies showed that *ATIC* c.347C>G polymorphism might be associated with MTX toxicity in Caucasians, compared to Asian patients. This scientific finding remains uncertain and tends to be associated with a higher frequency of the allele in Caucasians (Lee and Bae, 2016).

### 3.2 Azathioprine

AZA is an immunosuppressant used in managing SLE as second-line treatment. In the liver, AZA is initially converted to 6-mercaptopurine (6-MP) and then passes through three metabolism pathways. In the first pathway, 6-MP can be metabolized into 6-methylmercaptopurine (6-MMP) by thiopurine methyltransferase (TPMT). In the second pathway, 6-MP is oxidized by Xanthine Oxidase (XO) into 6-Thiouric Acid (6-TUA) which is an inactive metabolite. In the third pathway, metabolic processes of mercaptopurine nucleotide lead to the production of Thio inosine 5′-monophosphate (TIMP) by hypoxanthine phosphoribosyl transferase (HPRT). TIMP is converted by nudix hydrolase 15 (NUDT15) into thioguanine nucleotides (TGNs), including thioguanosine monophosphate (TGMP), thioguanosine diphosphate (TGDP), and thioguanosine triphosphate (TGTP). TGNs are the active metabolites of 6-MP which produce cytotoxic activity, specifically by inducing apoptosis in active T-cells. TGDP is converted to Thio deoxyguanosine triphosphate (TdGTP), which is subsequently incorporated into DNA. This process involves Thio deoxyguanosine diphosphate (TdGDP) as a metabolic intermediate. TGTP integrates into RNA disrupting the normal functions of these nucleic acids and leading to cell death (Mohamed et al., 2019; Fraenkel et al., 2021; Oglesby et al., 2013).

TIMP passes through alternative metabolic pathways and is transformed into 6-thio inosine 5′-triphosphate (6-TITP), a toxic metabolite. An enzyme called inosine triphosphate pyrophosphatase (ITPA) converts TITP into TIMP to restrict the accumulation. Another pathway is TIMP conversion into methyl thio inosine 5′-monophosphate (Me-TIMP) by TMPT. Me-TIMP has a role to impede the *de novo* synthesis of purine nucleotides, further compromising cellular processes crucial for T cell survival. Additionally, it inhibits Ras-related C3 botulinum toxin substrate, a protein essential in cell signaling pathways (Wright et al., 2004).

**FIGURE 2:**
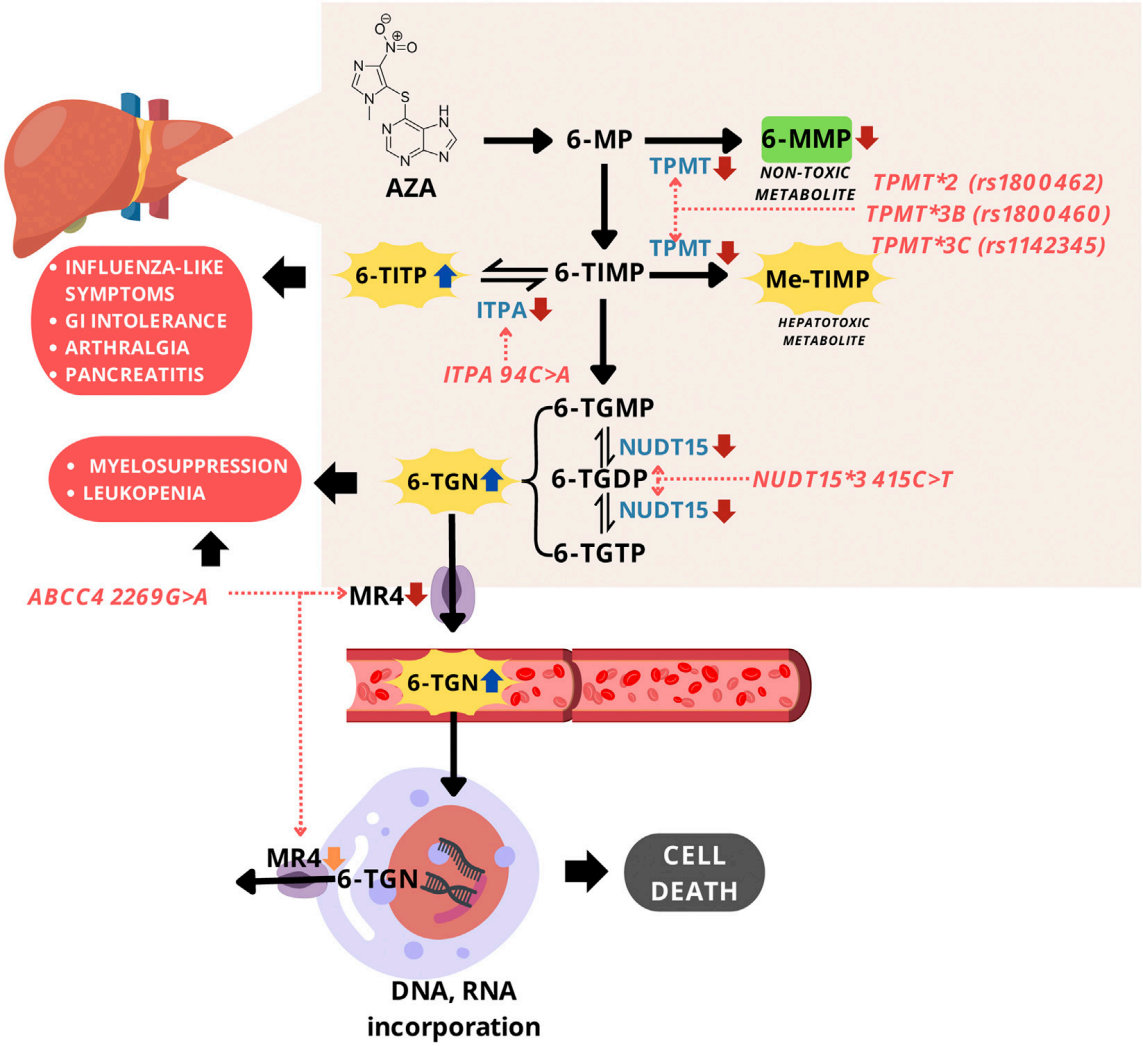
Mechanism of gene polymorphisms affecting AZA-related toxicity. Abbreviations: AZA, azathioprine; 6-MP, 6-mercaptopurine; 6-MMP, 6-methylmercaptopurine; 6-TIMP, 6-thioinosine 5'-monophosphate; 6-TITP, 6-thioinosine 5'-triphosphate; Me-TIMP, methyl-thioinosine 5'-monophosphate; 6-TGMP, 6-thioguanosine monophosphate; 6-TGDP, 6-thioguanosine diphosphate; 6-TGTP, 6-thioguanosine triphosphate; TGN, thioguanine nucleotides; TPMT, thiopurine S-methyltransferase; ITPA, inosine triphosphate pyrophosphatase; NUDT15, nudix hydrolase 15; MRP4, multidrug resistance-associated protein 4.

The use of AZA for SLE treatment is limited due to the drug-related toxicity reported, such as myelosuppression, leukopenia, pancreatic toxicity, and many more (Rashid et al., 2020; Liu et al., 2015; Fei et al., 2018b). The association between AZA toxicity and several SNPs has been investigated. The most common SNPs studied are *TPMT* gene polymorphisms [TPMT*2 G238C (rs1800462), TPMT*3B (rs1800460), and TPMT*3C (rs1142345)], *NUDT15* R139C, *ITPA* c.94C>A, and *ABCC4* c.2269G>A. Figure 2 shows effects of these gene polymorphisms on AZA-related toxicity.

#### 3.2.1 TPMT polymorphisms

*TPMT* gene is responsible for encoding thiopurine S-methyltransferase (TPMT), an enzyme that contributes to thiopurine drugs metabolism in cells. TPMT catalyzes the S-methylation of thiopurine including AZA into inactive and non-toxic forms. There are several alleles of the *TPMT* gene (TPMT*2 to TPMT*20) which affect TMPT activity, but the most frequently studied polymorphisms associated with AZA toxicity are TPMT*2 c.238G>C (rs1800462), TPMT*3B c.460G>A (rs1800460), and TPMT*3C c.719A>G (rs1142345). TPMT*2, TPMT*3B, and TPMT*3C are more common in Caucasians and Africans, while TPMT*3C is the most frequent in the Asian population (Gu et al., 2023). TPMT*2 c.238G>C were reported to prompt the substitution of alanine to proline. TPMT*3B c.460G>A and TPMT*3C c.719A>G are responsible for the deficiency or the loss of TPMT activity leading to AZA-related toxicity because of a decrease in the drug metabolism (Figure 2) (Steponaitiene et al., 2016). The reduction of AZA metabolism causes excessive accumulation of cytotoxic compounds, TGNs, inducing severe toxicity such as bone marrow toxicity and GI manifestations (Murugesan et al., 2009).

The data reported for the association of *TMPT* alleles and AZA-induced hepatotoxicity were conflicting. A meta-analysis showed that *TPMT* variations were not associated with hepatotoxic (Liu et al., 2015). Other studies found a higher risk of hepatotoxicity due to these polymorphisms, hence individualized AZA dosing could minimize the risk (Sheu et al., 2022). There was no correlation between the *TPMT* genotype and leukopenia incidence (p = 0.95) in Chinese autoimmune patients (Fei et al., 2018a). In the case of myelotoxicity, the data reported were conflicting because the rate of myelosuppression was significantly higher in Chinese patients with TPMT*2 than TPMT*3B and TPMT*3C polymorphisms, which could lead to clinical failure of AZA treatment (Fei et al., 2018a). Significant relationship was not observed between myelotoxicity and the *TPMT* polymorphisms (p = 0.973) in 70 Chinese patients receiving AZA (Su et al., 2020). Therefore, the presence of TPMT polymorphisms alone may not serve as a universally reliable predictor for AZA-induced myelotoxicity, particularly in certain populations such as East Asians, where additional genetic variants (e.g., *NUDT15*) have shown greater clinical relevance.

#### 3.2.2 NUDT15 polymorphism

*NUDT15* gene encodes NUDT15 enzymes, which function to dephosphorylate thiopurine triphosphate to monophosphate. This is included among the metabolism pathways of thiopurine drugs, such as AZA. The most common alleles studied and found to have an association with AZA-related toxicity consisted of NUDT15*3. This variant c.415C>T (rs116855232) causes the change of arginine to cysteine at position 139 and leads to function loss of NUDT15 enzyme due to a decrease in the thermal stability (Yang et al., 2014). The loss of NUDT15 function induces the excessive amount of thiopurine triphosphate and increases the number of TGNs incorporated into DNA and RNA, leading to severe AZA-related cytotoxicity. Among all polymorphisms related to AZA toxicity, NUDT15*3 was reported to have the strongest association with myelosuppression and leukopenia in patients, specifically Asians (Song et al., 2014; Martusevich et al., 2020; Wang et al., 2022). This is due to the frequency of the allele being higher in Asians population than in Caucasians (Tanaka and Saito, 2021). The genetic screening of NUDT15*3 gene has been implemented for personalizing AZA doses to decrease the risk of AZA-induced leukopenia in China (Wang et al., 2022).

#### 3.2.3 ITPA polymorphism

*ITPA* gene encodes ITPA which contributes to thiopurine metabolism, such as NUDT15 and TPMT enzymes. This has a role in preventing toxicity by restricting the accumulation of toxic thiopurine metabolites, 6-thioinosine-5-triphosphate, through conversion to TIMP (Nagamine et al., 2012). Gene variant of interest is *ITPA* c.94C>A (rs1127354), a missense mutation reported to be responsible for the decrease or even the loss of ITPA enzymatic activity (Cao and Hegele, 2002; Sumi et al., 2002). This impairs AZA metabolism process, leading to toxicity in patients with SLE and other diseases requiring AZA treatment (Nagamine et al., 2012). Compared to *TPMT* variants and *NUDT*15, *ITPA* c.94C>A was not associated with myelosuppression and hepatotoxicity (Steponaitiene et al., 2012; Chen et al., 2019), but influenza-like symptoms, digestive intolerance, pancreatitis, and arthralgia (Nagamine et al., 2012; Chen et al., 2021). The exact reason and mechanism of the phenomena remain inconclusive.

#### 3.2.4 ABCC4 polymorphism

*ABCC4* gene is responsible for encoding MRP4, an ATP-binding cassette transporter that functions as a transmembrane efflux pump to transfer 6-TGN out of the cell. *ABCC4* c.2269G>A (rs3765534) variant is suspected to be related to the side effects of leukopenia in patients given thiopurine because of 6-TGN accumulation (Song et al., 2014; Martusevich et al., 2020). *MRP4/ABCC4* c.2269G>A (rs3765534) decreases MRP4 function, might be responsible for myelosuppression (Milosevic et al., 2018), and is rarely found in the Caucasian race. However, the allele has been found at a higher frequency among the Asian population, including 14.7%–23% in the Japanese and 8.3% in the Han Chinese (Juster-switlyk et al., 2017; Zhao et al., 2016; Campbell et al., 2016).

### 3.3 Cyclophosphamide

**FIGURE 3:**
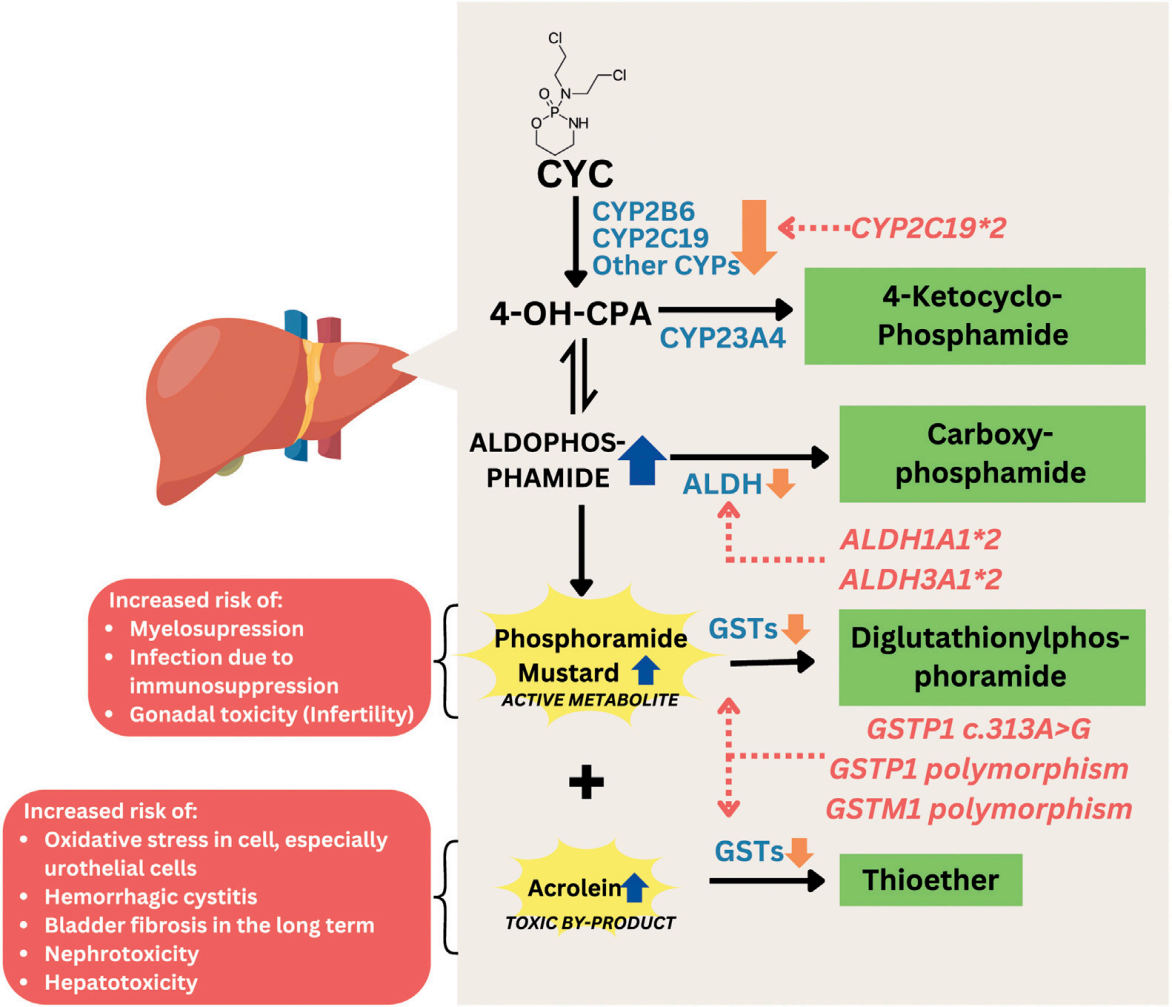
Mechanism of gene polymorphisms affecting CYC-related toxicity. Abbreviations: CYC, cyclophosphamide; CYP, cytochrome P450; 4-OH-CPA, 4-hydroxycyclophosphamide; ALDH, aldehyde dehydrogenase; GST, glutathione S-transferase.

CYC is a well-established alkylating agent widely used in the treatment of severe manifestations of SLE, particularly lupus nephritis (LN) (Zhang et al., 2014). Its efficacy lies in its potent immunosuppressive activity, which involves the inhibition of DNA replication and induction of cell death in rapidly proliferating immune cells. CYC is a prodrug that undergoes hepatic conversion to its active metabolite, 4-hydroxycyclophosphamide (4-OH-CPA), primarily facilitated by cytochrome P450 (CYP) enzymes, including CYP2B6, CYP2C9, and CYP3A4, with additional contributions from CYP2A6, CYP2C8, and CYP2C19 (Lamba et al., 2014). 4-OH-CPA is the major circulating metabolite and is in equilibrium with aldophosphamide, which subsequently breaks down into phosphoramide mustard, the active cytotoxic compound, and acrolein, a toxic byproduct responsible for bladder toxicity. While CYP enzymes are responsible for activation, enzymes such as glutathione S-transferase (GST) contribute to the detoxification of reactive metabolites, particularly acrolein (Alnasser, 2025). The active alkylating component, phosphoramide mustard, forms alkyl adducts with DNA through a phosphoramide aziridinium intermediate, while DNA alkylation induces a damage leading to cell death (Juster-switlyk et al., 2017). However, the use of CYC is frequently limited by its substantial adverse drug reactions. Well-documented CYC-related adverse effects include myelosuppression, urotoxicity (e.g., hemorrhagic cystitis), gonadotoxicity (e.g., ovarian failure and infertility), hepatotoxicity, and secondary malignancies (Mok, 2016; Mok et al., 1998). The occurrence and severity of CYC-related adverse effects can vary markedly among individuals, even with similar dosing regimens. It is increasingly recognized to be influenced by genetic differences, particularly single nucleotide polymorphisms (SNPs) in genes encoding CYC-metabolizing enzymes, transporters, and detoxification proteins. Genetic polymorphisms can affect the formation and clearance of both therapeutic and toxic CYC metabolites, ultimately altering CYC’s safety and efficacy profile. Polymorphisms in *GST* (e.g., *GSTM1* and *GSTP1*), *CYPs*, and aldehyde dehydrogenase (*ALDH*) genes have been associated with altered metabolism and increased risk of CYC-induced adverse effects in SLE patients (Illustrated in Figure 3) (Audemard-Verger et al., 2016; Indrawijaya et al., 2023; Hajdinák et al., 2020; Indrawijaya et al., 2024).

#### 3.3.1 GST polymorphisms

*GST* genes encode glutathione S-transferase (GST), an important enzyme involved in the detoxification of cyclophosphamide metabolites. GST catalyzes the conjugation of reactive metabolites, such as phosphoramide mustard, into less toxic compounds like 4-glutathionyl-cyclophosphamide (Dirven et al., 1994). A reduction or loss of GST activity can lead to the accumulation and prolonged exposure to these toxic metabolites, thereby increasing the risk of adverse effects and CYC-related toxicity (Hajdinák et al., 2020; Conklin et al., 2015). The well-known *GST* genes variants associated with occurrence of adverse effects-related to CYC are *GSTM1*, *GSTP1* and *GSTT1* (Audemard-Verger et al., 2016; Hahn et al., 2010). *GSTA1* is not included in this discussion, as its polymorphisms have been reported to influence the efficacy of CYC treatment rather than its toxicity or adverse effects (Indrawijaya et al., 2024; Wang et al., 2015).

*GSTM1* gene encodes Glutathione S-transferase Mu 1 (GSTM1), a key phase II detoxification enzyme that catalyzes the conjugation of glutathione to electrophilic compounds, including toxic metabolites of cyclophosphamide such as acrolein and phosphoramide mustard (NCBI, 2025a) *GSTM1* null genotype is the common polymorphism in *GSTM1*. Individuals with this genotype do not produce functional GSTM1, leading to impaired detoxification capacity and accumulation of acrolein and phosphoramide mustards. There is a strong evidence that the *GSTM1 null* genotype increases the risk of adverse effects from cyclophosphamide, regardless of other patient characteristics (age, gender, kidney function, and total CYC dose), with the odds ratio was 3.345, compared to those with functioning *GSTM1 gene* (Audemard-Verger et al., 2016). While some studies have reported a significant association between the *GSTM1* null genotype and increased CYC-related adverse effects, particularly when adjusted for clinical variables, others have found no relationship in the context of short-term high-dose regimens (Zhong et al., 2006). These discrepancies may reflect differences in study design, treatment protocol, population genetics, or definitions of toxicity.

*GSTP1* gene encodes glutathione S-transferase Pi 1, an enzyme involved in the phase II detoxification of reactive drug metabolites, including those generated during cyclophosphamide (CYC) metabolism. GSTP1 catalyzes the conjugation of electrophilic CYC byproducts with glutathione, facilitating their elimination (NCBI, 2025b; Hayes et al., 2005). A commonly studied variant of this gene is Ile105Val polymorphism (rs1695), which involves a single nucleotide change from A to G, resulting in the substitution of isoleucine (Ile) with valine (Val) at codon 105 of the enzyme (Hasni et al., 2016). This amino acid change alters the structure and function of GSTP1, potentially reducing its catalytic efficiency (Gorukmez et al., 2016). As GSTP1 is only active in its dimer form, both heterozygous and homozygous variant causes reduction in GTSP1 activity, causing an increase of acrolein and phosphoramide mustard concentration. Although increased levels of phosphoramide mustard are associated with improved cyclophosphamide efficacy in cancer patients, in SLE patients this increase has been linked to a higher incidence of myelotoxicity, particularly at higher cyclophosphamide doses. In contrast, gastrointestinal (GI) toxicity tends to be more frequent at lower doses of cyclophosphamide in SLE, likely due to its non-linear pharmacokinetic profile, which results in greater biliary excretion of toxic metabolites following low-dose administration compared to high-dose administration (Zhong et al., 2006).

*GSTT1* gene encodes for the enzyme glutathione S-transferase theta 1, which also plays a role in the detoxification of toxic metabolites of CYC. A common polymorphism involves a homozygous deletion of the *GSTT1* gene, referred to as the *GSTT1* null genotype, resulting in the absence of functional enzyme activity. While the *GSTT1* null genotype has been associated with increased chemotherapy-related toxicities in oncology settings (Cho et al., 2010; Wang et al., 2016; Aguiar et al., 2012), this variant does not appear to significantly affect the risk of cyclophosphamide-related adverse effects—such as myelosuppression and gastrointestinal toxicity—in SLE patients (Zhong et al., 2006). This may be due to higher, more frequent doses of CYC, and different patient characteristics in cancer patients compared to SLE patients.

#### 3.3.2 CYP polymorphisms

*CYP* genes are responsible for encoding a family of Cytochrome P450 enzymes which activate CYC to the active metabolite, 4-OH-CPA. It is generated mainly by CYP2B6, CYP3A4, and CYP2C9, with additional contributions from CYP2C19, CYP2A6, and CYP2C8. Genetic polymorphisms in these enzymes can significantly influence CYC’s pharmacokinetics, leading to interindividual differences in efficacy and toxicity. Excessive amounts or prolonged duration of active metabolites exposure to body cells may be associated with CYC-related adverse effects, such as ovarian toxicity. Among these enzymes, the most studied polymorphism variants are *CYP2B6,* which has been studied in relation to CYC metabolism and toxicity. Three notable variants of *CYP2B6* include c.516G>T (rs3745274), c.785A>G (rs2279343), and −750T>C (rs4802101). The c.516G>T variant, which defines the CYP2B6*6, results in the substitution of glutamine with histidine at codon 172 (Q172H), leading to reduced enzyme expression and enzyme (Abuelsoud et al., 2021). The c.785A>G (rs2279343) variant causes a lysine-to-arginine substitution at position 262 (K262R) and often co-occurs with c.516G>T as part of the CYP2B6*6, further contributing to impaired metabolic activity (Tran et al., 2008). This may reduce the formation of active metabolites, potentially lowering toxicity but also decreasing efficacy. Meanwhile, the −750T>C (rs4802101) variant is located in the promoter region, which may influence gene expression levels, though its clinical impact remains inconclusive (Zanger and Klein, 2013). For instance, a study involving 116 Chinese patients, individuals carrying the C allele had a significantly lower incidence of gastrointestinal toxicity and leukocytopenia compared to those with the wild-type TT genotype (Shu et al., 2016). However, other studies have reported that there was no association between *CYP*2*B6* polymorphisms and reduced CYC-related adverse effects (Kumaraswami et al., 2017; Singh et al., 2007).

CYP3A4 is another major to CYC metabolism. One notable polymorphism of *CYP3A4* is CYP3A4*1B, characterized by an A>G transition in the 5′-flanking region of the gene. This variant has been associated with altered gene expression and enzymatic activity (Su et al., 2010). While there is still no study investigating the functional impact of this variant on CYC-related adverse effects in SLE patients, a study on cancer patients found no significant association between CYP3A4*1B and the pharmacokinetics of CYC or its active metabolite, 4-OH-CPA (Ekhart et al., 2008). CYP2C9 also plays a role in cyclophosphamide (CYC) metabolism, but contributes only minimally to its activation into 4-hydroxycyclophosphamide (4-OH-CPA) (Griskevicius et al., 2003).

CYP2C19, CYP2A6, and CYP2C8 play ancillary roles in CYC metabolism (Muñiz et al., 2022). Among these, the CYP2C19*2 is one of the most extensively studied variants due to its high frequency in many populations, especially in Asian population, and its association with reduced enzymatic activity (Audemard-Verger et al., 2016). The CYP2C19*2 c.681G>A (rs4244285) in exon 5 is responsible for a decrease in CYP enzyme activity. There are conflicting data regarding whether *CYP2C19* is associated with a higher risk of CYC toxicity. Several studies stated that the presence of this allele is related to ovarian toxicity and the risk of CYC treatment failure (Kumaraswami et al., 2017; Lee et al., 2016; Ngamjanyaporn et al., 2011). However, a study associated CYP2C19*2 presence with a lower risk of toxicity, specifically ovarian toxicity in Indian patients (Singh et al., 2007). Another study reported that CYP2C19*2 had no association with CYC-related toxicity (Audemard-Verger et al., 2016). Further investigation is required to understand more about the reason for these results. Additionally, studies on genetic polymorphisms of *CYP2A6* and *CYP2C8* in the context of CYC metabolism are limited, particularly in patients with systemic lupus erythematosus (SLE). Thus, their clinical relevance in modulating CYC efficacy or toxicity remains unclear and warrants further investigation.

#### 3.3.3 ALDH polymorphisms

*ALDH* encodes Aldehyde Dehydrogenase (ALDH), an enzyme that contributes to CYC metabolism and detoxification, such as GST. ALDH converts aldophosphamide to a non-toxic metabolite, carbophosphamide. The presence of two variants (ALDH3A1*2 and ALDH1A1*2) is known to reduce the activity of the ALDH enzyme, which affects the detoxification capacity of cyclophosphamide (CYC), theoretically increasing the risk of toxicity from CYC (Ekhart et al., 2008). A study involving 113 Caucasian patients receiving high-dose chemotherapy with a combination of CYC, thiotepa, and carboplatin showed that the ALDH1A1*2 variant, located in the promoter region and potentially having significant gene regulatory effects, was associated with an increased risk of liver toxicity and hemorrhagic cystitis (Ekhart et al., 2008). Another study at Michigan Hospital involving 846 patients receiving CYC-based chemotherapy regimens indicated that the presence of the *ALDH1A1* c.1234A>G (rs8187996) variant was actually associated with a reduced risk of ≥3 toxicity or the need for treatment modification due to toxicity. The rs8187996 variant is located in the intron (non-coding) region, which has a minor effect on gene regulation (Hwang et al., 2022). The differences in the location and functional effects of the genetic variants studied, as well as the differences in chemotherapy regimens used, may explain the differing results between the two studies.

### 3.4 Mycophenolate mofetil

**FIGURE 4:**
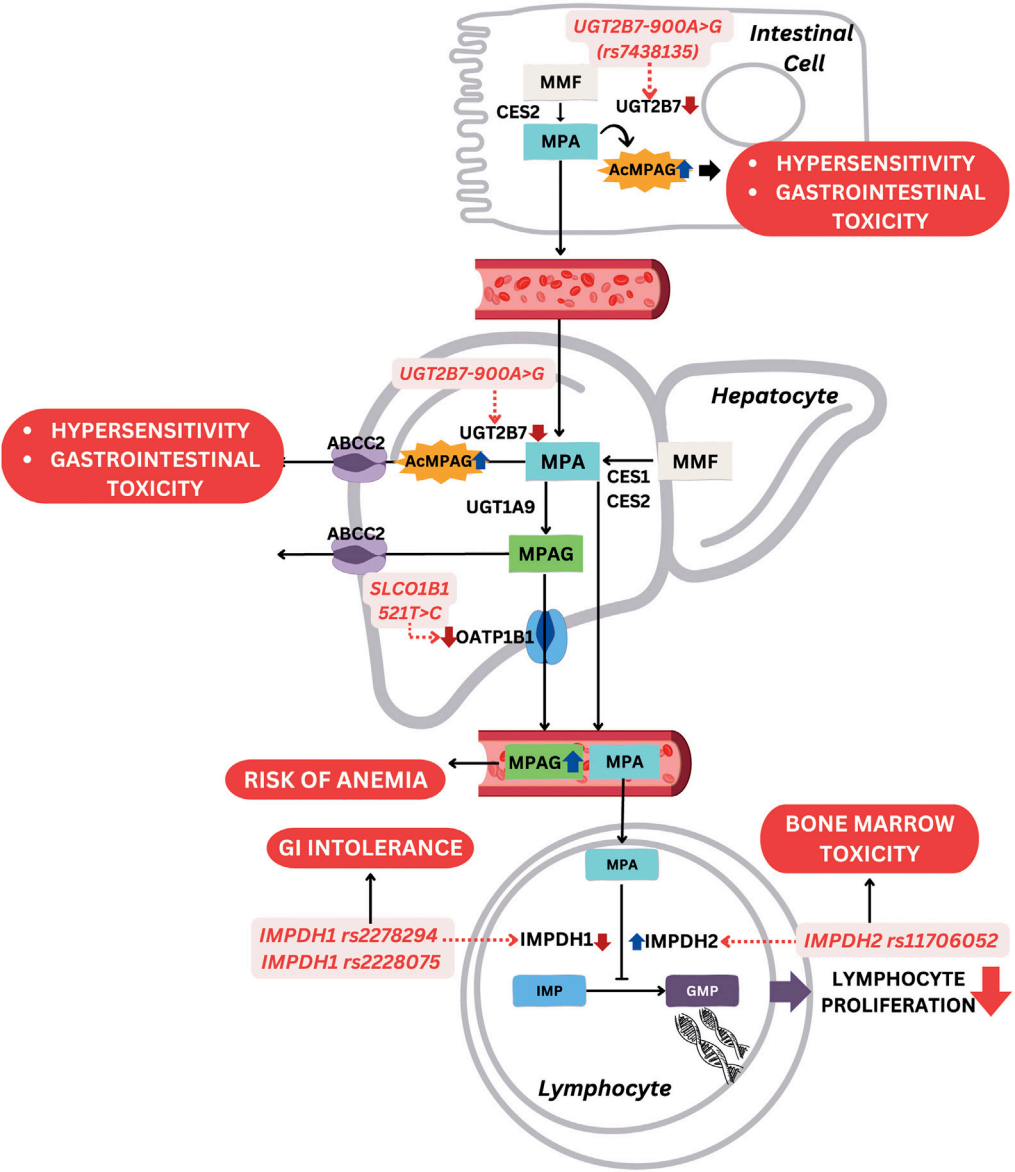
Mechanism of gene polymorphisms affecting MMF-related toxicity. Abbreviations: MMF, mycophenolate mofetil; MPA, mycophenolic acid; AcMPAG, acyl-glucuronide of mycophenolic acid; MPAG, mycophenolic acid 7-O-glucuronide; IMP, inosine monophosphate; GMP, guanosine monophosphate; CES2, carboxylesterase 2; UGT, UDP-glucuronosyltransferases; ABCC2, ATP-binding cassette sub-family C member 2; OAT, organic anion transporters; SLCO1B1, solute carrier organic anion transporter family member 1B1; IMPDH, inosine 5'-monophosphate dehydrogenase; GI, gastrointestinal.

MMF is a pro-drug for mycophenolic acid (MPA), which hinders inosine monophosphate (IMP), thereby suppressing the production of guanosine monophosphate (GMP) to initiate reduced proliferation of B and T cells, as well as diminished production of antibodies. This pro-drug is used to treat SLE, specifically LN, due to the immunosuppressive activity. MMF experiences rapid absorption in the GI system and is transformed into MPA by esterase enzymes, particularly carboxylesterase 2 (CES2). MPA then engages in an enterohepatic cycle facilitated by an organic anion-carrying polypeptide. In this cycle, glucuronidation transforms MPA into the inactive forms, namely, 7-O-glucoside and acyl-glucuronide. The liver, kidney, and GI tract use UDP-glucuronosyltransferase 1A9 (*UGT1A9*) and other UGT1A superfamily enzymes for this glucuronidation process, leading to the formation of MPA 7-O-glucuronide (MPAG). The pharmacologically active metabolite, acyl-glucuronide of MPA (AcMPAG), is thought to contribute to the typical adverse effects of MMF. Following glucuronidation, MPAG is eliminated from the body through organic anion transporters (OATs) (Liu et al., 2015). Several MMF-related toxicities have been reported, such as GI manifestation, infections, anemia, low platelet count, leukopenia, and many more (Mok, 2015; Riskalla et al., 2003). The most common gene polymorphisms associated with MMF toxicity are *IMPDH*, *UGT2B7*, and *SLCO1B1* polymorphisms. For example, *IMPDH2* c.3757T>C (rs11706052) has been associated with gastrointestinal toxicity (OR = 3.05, 95% CI: 1.22–7.60, *p* = 0.02), *UGT2B7* -900A>G (rs7438135) with GI toxicity (OR = 2.34, 95% CI: 1.14–4.79, *p* = 0.02), and *SLCO1B1* c.521T>C (rs4149056) with hematologic toxicity (OR = 3.10, 95% CI: 1.23–7.82, *p* = 0.02) (Na Takuathung et al., 2021). Figure 4 shows effects of these gene polymorphisms on MMF-related toxicity.

#### 3.4.1 IMPDH polymorphisms

*IMPDH* encodes an enzyme called inosine 5′-monophosphate dehydrogenase 1 (IMPDH), which functions to produce the guanosine required in the lymphocytes proliferation process. MPA works by inhibiting IMPDH activity, while *IMPDH1* and *IMPDH2* variants influence the incidence of MMF toxicity. The variant of *IMPDH1* -106C>A (rs2278294) and c.1575G>A (rs2228075) were strongly associated with infection susceptibility and GI intolerance, such as nausea, vomiting, and diarrhea (Ohmann et al., 2010; Shu et al., 2021). *IMPDH1* encodes inosine monophosphate dehydrogenase type I, a key enzyme involved in the *de novo* synthesis of guanine nucleotides, which is essential for the proliferation of T and B lymphocytes. Mycophenolic acid (MPA), the active metabolite of mycophenolate mofetil (MMF), exerts its immunosuppressive effects by inhibiting this enzyme. Certain *IMPDH1* polymorphisms, such as nonsynonymous variants including rs2278294 and rs2228075, have been reported to reduce enzymatic activity through altered protein stability and impaired tetramer formation, without significantly affecting mRNA levels, indicating a post-translational regulatory mechanism. This reduction in IMPDH1 activity may enhance the pharmacodynamic effects of MPA, thereby intensifying immunosuppression and predisposing patients to infection. Additionally, enhanced local MPA effects in the gastrointestinal tract may contribute to increased risk of nausea, vomiting, and diarrhea. Ethnic differences in the distribution of IMPDH1 haplotypes may also partially explain interindividual variability in toxicity profiles (Wu et al., 2010). Meanwhile, *IMPDH2* c.3757T>C (rs11706052) was associated with bone marrow toxicity due to an increase in IMPDH activity (Neerman and Boothe, 2003).

#### 3.4.2 UGT2B7 polymorphisms

*UGT* encodes UDP-glucuronosyltransferases (UGTs), such as *UGT1A9* and *UGT1A8*, which convert MPA to the inactive MPAG. However, one variant of UGT known as *UGT2B7* produces a minor highly reactive metabolite, AcMPAG, which is associated with drug toxicity (Bernard et al., 2006). A *UGT2B7* variant called *UGT2B7 -*900A*>*G (rs7438135) was associated with a higher risk of leukopenia and anemia. In this variant, adenine (A) at coding DNA position 900 is replaced by guanine (G), causing a decrease in *UGT2B7* activity and leading to an accumulation of toxic metabolite, AcMPAG. Another genetic variant, *UGT2B7* c.802C>T (rs7439366), was identified with contributions to increased susceptibility to infections, particularly *Pneumocystis carinii* pneumonia (Shu et al., 2021).

#### 3.4.3 SLCO1B1 polymorphism

*SLCO1B1* or solute carrier OATs family member 1B1, is a gene that encodes a membrane transporter protein primarily found in the liver. This transporter plays a crucial role in facilitating various endogenous and exogenous substances, including MMF, across cell membranes. *SLCO1B1* participates in the clearance of numerous drugs, and the most common alleles studied include *SLCO1B1* c.521T>C (rs4149056) that causes the substitution of valine to alanine at position 174. The substitution leads to a decrease in the ability of the transporter to facilitate MPA intake, increasing MPA plasma concentration. S*LCO1B1* c.521T>C was associated with MMF-induced anemia (Shu et al., 2021), but a previous study did not find a relationship between this polymorphism and toxicity (Neerman and Boothe, 2003).

## 4 Discussion

### 4.1 Immunosuppressant-related toxicity

Immunosuppressant is used when the disease progresses from moderate to severe condition and the administration of GCs as SLE first-line treatment cannot sustain clinical remission (Gatto et al., 2019). In general, immunosuppressant mechanism of action requires suppressing and decreasing the autoimmune responses, which can target various organs and systems in the body. This serves to minimize damage in various organs, thereby preventing life-threatening conditions. The use of immunosuppressant is based on the organs engaged in SLE activity and the conditions of patients. Moderately active lupus and joints involved are treated with MTX, while LN and other severe cases are treated primarily with MMF and CYC (Mohamed et al., 2019; Fraenkel et al., 2021). Immunosuppressants contribute to reduced GC exposure, improved disease stabilization, and enhanced long-term survival. Nonetheless, their use is associated with a high incidence of adverse effects, with reported toxicity rates ranging from 42.8% to 97.3%. Common toxicities include infections, gastrointestinal disturbances, amenorrhea, ovarian dysfunction, hematologic cytopenia, hepatic dysfunction, bone marrow suppression, pulmonary toxicity, and others. The specific toxicity profile often depends on the pharmacological agent used and the patient’s genetic predisposition (Oglesby et al., 2013).

### 4.2 Pharmacogenomics and personalized treatment

The integration of pharmacogenomic strategies into SLE management—particularly regarding immunosuppressive therapy—has gained increasing attention. Advances in pharmacogenetic research have identified single nucleotide polymorphisms (SNPs) as critical determinants of interindividual variability in drug response and toxicity (Meng et al., 2018). Genetic polymorphisms in genes involved in drug metabolism and enzymatic activity—such as TPMT*2, *3B, *3C variants in azathioprine (AZA) recipients and *MTHFR* c.667C>T and c.1298A>C in MTX-treated patients—have been associated with increased susceptibility to drug-induced toxicity (Van Der Put et al., 1998; Gu et al., 2023; Murugesan et al., 2009) (see Table 1).

Furthermore, ethnic variability contributes significantly to the genetic landscape of SLE, affecting the distribution and impact of pharmacogenomic markers. For instance, the *ATIC* c.347C>G polymorphism has shown differential toxicity outcomes across racial groups in MTX users (Lee and Bae, 2016). Similarly, the *ABCC4* c.2269G>A variant has been linked to AZA toxicity, with the highest incidence observed in Asian populations and minimal occurrence in Caucasians (Juster-switlyk et al., 2017; Zhao et al., 2016; Campbell et al., 2016; Milosevic et al., 2018). These findings underscore the need for population-specific pharmacogenomic research to develop equitable and evidence-based treatment strategies across diverse demographic groups.

### 4.3 Current research gaps and future directions

Current pharmacogenomic studies in SLE predominantly focus on single-drug and single-gene associations, with limited exploration into polygenic interactions or the effects of gene–drug combination therapies on toxicity profiles. Broader research encompassing gene-gene and gene-environment interactions is essential to fully understand the complexity of treatment responses in SLE. The incorporation of advanced genomic technologies such as next-generation sequencing (NGS) holds promise for generating more comprehensive datasets. Such data could inform clinical decision-making and facilitate the development of personalized medicine approaches tailored to the genetic and clinical characteristics of individual patients.

## 5 Conclusion

Understanding the association between immunosuppressant-related adverse effects and gene polymorphisms is crucial for assessing patient risk, enabling individualized drug therapy, and enriching global pharmacogenetic knowledge. Insights from pharmacogenetics can support the prediction and prevention of adverse reactions to drugs such as methotrexate (MTX), azathioprine (AZA), cyclophosphamide (CYC), and mycophenolate mofetil (MMF). Nevertheless, current evidence is limited by small sample sizes, underrepresentation of specific populations (e.g., pediatric and ethnically diverse groups), and methodological challenges in genotyping and data interpretation.
